# Understanding doula integration through nurses’ eyes: The triadic relationship in hospital-based maternity care

**DOI:** 10.1186/s12884-026-09629-6

**Published:** 2026-07-23

**Authors:** Leanne T. Burke, Ah-Taisa Banks, Griffin Chanley, Madison Jang, Candice Taylor Lucas, Yuqing Guo

**Affiliations:** 1 University of California, Irvine, Sue & Bill Gross School of Nursing, Irvine, CA USA; 2 Master’s Entry Program in Nursing, Student, University of California, Irvine, Sue & Bill Gross School of Nursing, Irvine, CA USA; 3 Department of Pediatrics, University of California, Irvine, School of Medicine, Irvine, CA USA

**Keywords:** Implementation science, Triadic relationships, Labor and delivery, Doulas, Perinatal nurses, Collaborative care, Interprofessional teamwork, Maternal health

## Abstract

**Background:**

Hospital-based doula programs remain rare (7.4% of US birthing hospitals), yet evidence supports continuous labor support for improved maternal-neonatal outcomes. Implementation challenges persist nationally despite policy initiatives. Few studies systematically examine perinatal nurses’ perspectives, despite them being key stakeholders whose attitudes critically influence program success. The objective of this study was to conduct a pre-implementation needs assessment identifying specific barriers, facilitators and contextual factors influencing hospital-based doula integration from perinatal nurses’ perspectives.

**Methods:**

Qualitative study with seven perinatal nurses at an urban academic medical center preparing to launch a doula program. Semi-structured interviews explored nurses’ experiences with and perceptions of doula integration. Inductive content analysis was conducted using Atlas.ti web version.

**Results:**

Participants were predominantly bachelor’s-prepared (71.43%), White (57.1%), aged 23–41 (median 32.5), with labor and delivery experience ranging from < 5 to 20 years. Most participants (71.4%) rarely worked with doulas, with 28.6% sometimes working with them. Analysis revealed three themes: (1) The Triadic Nurse-Patient-Doula Relationship – nurses consistently conceptualized doula integration as an interconnected three-party system (nurse-patient-doula) rather than separate dyadic relationships, (2) Stabilizing Factors that Strengthen Triadic Collaboration – open communication and relationship-building strengthened collaboration; (3) Destabilizing Factors that Undermine Triadic Collaboration – role and scope confusion, advocacy rigidity, mistrust and a lack of institutional infrastructure undermined collaboration.

**Conclusions:**

This needs assessment identified triadic relationship dynamics as the central factors affecting successful integration of doulas, representing a reframing of doula implementation as an organizational challenge inherent in implementation. Findings provide implementation recommendations including communication protocols, standardized training, role complementarity emphasis, and collaborative birth planning mechanisms. The triadic relationship offers practical guidance for healthcare leaders integrating doula support while addressing complex interpersonal dynamics influencing implementation outcomes. Future studies should include racially and ethnically diverse nursing perspectives related to implementation strategies.

## Introduction

Strong evidence shows that continuous labor support throughout the childbearing period (e.g. support provided by a doula), is associated with improved maternal and neonatal outcomes, including a decrease in cesarean sections, length of labor, use of pain medications like epidural anesthesia, improved Apgar scores, breastfeeding initiation and continuation [1, 2]. Recent evidence highlights that hospital-based doula programs remain rare nationwide [3]. This 2025 environmental scan of US birthing hospitals found that only 7.4% (range 2.2% − 17.3%) offered doula services, with notable regional variation, and inconsistent website language, making it difficult for patients to identify available support [3].

Despite robust evidence that continuous labor support by doulas improves maternal and neonatal outcomes - including cesarean rates, labor duration, and maternal dissatisfaction [2, 3] - hospital-based doula programs remain limited nationwide. Recent Centers for Disease Control (CDC) data reveal persistent disparities, with Black and Indigenous women experiencing maternal mortality rates three to four times higher than white women, underscoring the urgent need for equitable, evidence-based interventions [4]. While several states have begun Medicaid coverage for doula services, implementation challenges, such as funding, workforce diversity, and organizational readiness, hinder widespread adoption [5, 6]. Models like the Birth Sisters Program demonstrate that hospital-based doula support can promote health equity when integrated effectively, but systemic barriers, such as role ambiguity, reimbursement issues, and structural inequities, remain significant obstacles [5, 6] Addressing these multi-level barriers requires systematic stakeholder engagement and tailored strategies to ensure sustainable integration across diverse settings [7]. These findings underscore the persistent challenges of building sustainable, scalable doula programs in complex hospital environments, where funding limitations, diverse patient needs, workforce shortages, and geographic care deserts constrain equitable access [8, 9]. For example, California’s roll-out of the Medi-Cal doula benefit illustrates these barriers: despite increasing utilization, stakeholders report limited provider awareness, role ambiguity, complex credentialing, and strained nurse-doula communication, reflecting barriers documented nationally in hospital-based integration efforts [8, 10, 11].

Current implementation approaches often lack systematic needs assessments that identify specific stakeholder perspectives and contextual factors before launching programs [8, 9]. Experience shows that without understanding how key stakeholders view doula integration, especially nurses who serve as the primary care coordinators, programs may address organizational barriers but overlook critical interpersonal and workflow challenges that determine daily implementation success [10, 12, 13]. When doula programs fail, it’s often due to workplace tensions and role confusion rather than lack of evidence for effectiveness. Yet few studies have asked nurses, who coordinate moment-to-moment care at the bedside, what would help doula integration succeed in their daily practice [10, 12].

Our study aimed to close this gap by conducting a pre-implementation needs assessment of perinatal nurses’ perspectives on doula integration, identifying specific barriers, facilitators, and contextual factors that influence implementation success. We sought to understand which key factors, identified by nurses who coordinate patient care at the bedside, would either support or hinder successful doula integration. By asking nurses before implementation rather than after, we could proactively design a program that addresses their concerns and leverages their expertise. Our study aligns with the Consolidated Framework for Implementation Research (CFIR) that emphasizes engaging stakeholders early in the process to maximize program success and sustainability [7].

## Methods

### Design

To gain an in-depth understanding of the contextual factors influencing doula integration into perinatal departments, a qualitative methodology was selected to explore perinatal nurses baseline experiences and perspectives, and to analyze factors affecting successful integration. The study was conducted at an urban academic medical center preparing to launch a doula program, capturing nurses’ perspectives during the critical pre-implementation phase when stakeholder attitudes significantly influence adoption success [5]. The interdisciplinary research team included an experienced perinatal nurse and qualitative researcher (first author), a nurse scientist (last author), a physician (co-author) and three graduate nursing students with formal training in qualitative methods. This composition provided both insider knowledge of perinatal nursing practice and analytical distance necessary for rigorous qualitative inquiry. The interview guide (See Table 1) was informed by existing literature on nurse-doula collaboration [10, 12, 13], implementation barriers [7, 9, 10, 13], and interprofessional teamwork in maternity settings [10, 12, 13]. The first two interviews served as pilot, after which minor adjustments were made to question ordering and probe specificity. No substantial content changes were needed, and these interviews were included in the final analysis as they yielded rich, relevant data. Team members engaged in reflexivity exercises to identify potential biases and assumptions about doula integration prior to data collection and analysis. Peer debriefing sessions during analysis allowed for consensus-building during the coding process to ensure interpretations were clear and agreed upon by all.

**Table 1:** Semi-structured interview guide with supporting literature

Domain	Primary Question	Follow-up Probes	Supporting Literature
Experiences with Doulas	Can you tell me about your experiences (perspectives or feelings) when working with doulas?	• Tell me about a time when you cared for a patient with a doula that was very meaningful.	*Neel et al.*,* 2019 *[10] *Roth et al.*,* 2016 *[12] *Myrick et al.*,* 2024 *[13]
Impact on Patient Care	How has a patient having a doula impacted your interactions when caring for your patients? *Or if they have no experience with doulas*: How do you perceive doulas impacting your interactions when caring for your patients?	• Can you share some examples you have observed (or might expect) (positives or challenges) in the interactions between doulas, other healthcare providers, and the patient during the labor and birth process?	*Neel et al.*,* 2019 *[10] *Gebel et al.*,* 2024 *[9] *Myrick et al.*,* 2024 *[13] *Marshall et al.*,* 2024 *[28]
Communication & Coordination	Describe how you approach (or would approach) communication and care coordination when working with a doula to ensure optimal support and continuity of care for the patient.	• Can you describe some examples of effective means of communication and care coordination? • What are some challenges and concerns of working along with doulas to support the mother? Feel free to provide specific examples.	*Neel et al.*,* 2019 *[10] *Myrick et al.*,* 2024 *[13] *Gebel et al.*,* 2024 *[9] *Damschroder et al.*,* 2009 *[7]
Team Integration	What essential knowledge, skills, and/or competencies would you want doulas to strengthen to be effective members of an interprofessional team at your facility?	[Open-ended, no specific probes]	*Neel et al.*,* 2019 *[10] *Roth et al.*,* 2016 *[12] *Myrick et al.*,* 2024 *[13]
Patient Selection	What types of patients do you believe would benefit the most from doula care?	[Open-ended, no specific probes]	*Gebel et al.*,* 2024 *[9] *Marshall et al.*,* 2024 *[28]

Interview questions were developed based on existing literature addressing nurse-doula collaboration, implementation barriers, and interprofessional teamwork in maternity settings. The Consolidated Framework for Implementation Research (CFIR) informed questions about organizational and team-level factors affecting doula integration

## Procedure

Inclusion criteria required participants to be over 18 years old, English-speaking, registered nurses currently working in the perinatal departments (Labor and Delivery, Antepartum, and Mother/Baby units) at the study site. Such criteria were selected to capture variation in nurse perspectives across experience levels, units, and prior doula exposure. Labor and delivery nurses have the most direct doula interactions during active birth, while Antepartum and Mother/Baby nurses are present during the times before and after childbirth, thus they may have different implementation concerns based upon their clinical roles.

Following University Institutional Review Board approval, recruitment occurred through perinatal department staff meetings, flyers posted throughout the department, and colleague referrals. Data collection took place between February and April 2024. Following the completion of seven interviews, the research team collectively reviewed all transcripts and reached consensus that data saturation had been achieved at the fifth interview, with no new themes or insights emerging during the sixth and seventh interviews. As additional recruitment was unlikely to yield new information, participant enrollment was concluded at that time [14, 15]. Of the 12 volunteers who expressed interest in the study, seven participated in the interviews (58% response rate). The five non-participants cited scheduling conflicts.

Participant demographics were collected prior to the interviews using a secure cloud-based survey system supported by the university where the researchers are affiliated. Semi-structured interviews using the interview guide were conducted via secure videoconferencing to accommodate nurse scheduling constraints and ensure confidentiality. The lead researcher conducted each interview to ensure consistency, the duration (30–45 min) was participant driven and based on response depth. Following informed consent, interviews began with general questions about doula interactions, progressing to specific examples and implementation considerations. All interviews were audio-recorded, stored in password-protected files, transcribed, and verified for accuracy against field notes.

### Data analysis

Transcriptions were imported to the web version of Atlas.ti and reviewed by the research team for inductive content analysis. Steps to this process included: (1) immersive reading of transcripts, (2) independent coding of all transcripts to identify the individual experiences and perspectives of the perinatal nurses interviewed and development of the codebook, (3) similar codes were distilled, refined and consolidated into categories with coding discrepancies being resolved through structured discussion sessions and consultation with the last author who has extensive experiences with qualitative design until 100% consensus was achieved with the entire team, and (4) final categories were sorted into the final themes. This analytical process involved the research team meeting regularly during the analysis phase to complete iterative cycles of independent coding followed by team consensus-building, through which the research team moved from initial codes to broader categories by identifying patterns and conceptual relationships across participants’ experiences. These categories were then synthesized into final themes that represented the key dimensions of nurse-doula collaboration [14, 16].

Trustworthiness was established to ensure rigor of qualitative data analysis through multiple strategies consistent with Lincoln and Guba’s [17] criteria: (1) credibility through triangulation of data sources (interviews, field notes, and demographic data), and peer debriefing sessions among research team members; (2) dependability and confirmability through maintenance of a comprehensive audit trail documenting analytical decisions, coding iterations, and consensus discussions, plus external audit by an experienced qualitative researcher not involved in data collection; and (3) transferability through thick description of context, participants, and findings.

## Results

Seven perinatal nurses from an academic medical center in southern California participated in this study. Most held bachelor’s degrees (five with BSN degrees and two with master’s degrees). Four identified as White, with one participant each identifying as Hispanic/Latino/a/e, Black/African American, and Asian, and one as Other (Note: participants had the option to select more than one identity option). Participants ranged in age from 23 to 41 years (median = 32.5). The majority of participants (*N* = 4) had 6–10 years of Labor and Delivery experience. Five participants (71.4%) rarely worked with doulas (See Tables 2 and 3). Nursing leadership from this medical center confirmed these demographics were indicative of the broader perinatal nursing staff.

**Table 2:** Participants’ Characteristics

Characteristics	*n* = 7*
Age (years)	
Range	23–41
Median	32.5
Race/Ethnicity*	
White	4
Hispanic/Latino/a/e	1
Black/African American	1
Asian	1
Other	1
Education	
BSN	5
MSN	2

*N = 8 for Race/Ethnicity. Note, participants had the option to select more than one identity option

**Table 3:** Participants’ clinical and doula experience

Participant Number	Clinical Experience and Doula Exposure
Participant #1	Labor & Delivery nurse (*≥* 10 years of experience), rare doula exposure
Participant #2	Labor & Delivery nurse (*≥* 10 years of experience), some doula exposure
Participant #3	Labor & Delivery nurse (1.5 years of experience), rare doula exposure
Participant #4	Labor & Delivery nurse (1.5 years of experience), rare doula exposure
Participant #5	Labor & Delivery nurse (6.5 years of experience), rare doula exposure
Participant #6	Labor & Delivery nurse (*≥* 10 years of experience), rare doula exposure
Participant #7	Labor & Delivery nurse (9 years of experience), some doula exposure

Inductive content analysis of interview transcripts identified 22 initial codes. Through iterative review and team discussion, codes were refined to 14 then grouped into eight categories based on conceptual similarities and relational patterns [14, 16]. Categories were further abstracted into three themes (See Table 4). The three themes are: The Triadic Nurse-Patient-Doula Relationship, Stabilizing Factors that Strengthen Triadic Collaboration, and Destabilizing Factors that Undermine Triadic Collaboration. Exemplary quotes were selected for each code and theme, with minor edits for readability (removal of filler words like ‘like’ and ‘um’) indicated by ellipses (See Table 5).

**Table 4:** Themes, categories, and representative codes

Theme (*n* = 3)	Categories (*n* = 8)	Final Codes (*repeated) (*n* = 14)
The Triadic Nurse-Patient-Doula Relationship	Nurse-Doula Dyad Nurse-Patient Dyad Doula-Patient Dyad	• Hierarchy/Role Boundaries • Impact of Doula on RN-Patient Relationship • Pre-existing Patient-Doula Bonds • Collaborative Teamwork*
Stabilizing Factors that Strengthen Triadic Collaboration	Open Communication Relationship Building and Support	• Collaborative Teamwork* • Shared Care Planning • RN Receptivity/ Appreciation • Advocacy & Emotional Support • Role Complementarity
Destabilizing Factors that Undermine Triadic Collaboration	Individual-Level Issues Interpersonal Tensions System-Level Gaps	• General Mistrust of Doulas • Scope of Practice/Training • Variable Doula Practices • Unclear RN Expectation of Doula Contributions • Advocacy for Unavailable Options • Advocacy Rigidity

**Table 5:** Selected Exemplar Quotes

Theme 1: The Triadic Nurse-Patient-Doula Relationship	
The Triadic Relationship	*“…communicating with the doula…before speaking to the patient like they’re more like a member of the healthcare team and then…formulating a plan together and then bringing it up…because the doulas are really great at being advocates for the mom and they’re very knowledgeable on the mom’s plan and sometimes staff can forget that…So if we were all on the same page and then bringing up to the mom together about our plan.”* Participant #5
Nurse-Doula Dyad	*“As nurses*,* we would have to be very well staffed in order to sit at the bedside and rub our patients back for as much as they really needed during labor. We have charting to keep up with; we have maybe more than one patient. We have documenting assessments*,* talking with the doctors*,* we’re helping each other. Another nurse is having trouble with their patient; we jump in and help them. So*,* for a patient to have somebody stay at their bedside and really give them 100% focus.”* Participant #2
	*“We (doula and RN) sat at the bedside for about 30 minutes just rearranging the pillows under her (patient’s) legs and the doula was so patient… we were bouncing (ideas) off each other.”* Participant #4
Nurse-Patient Dyad	*“She [the doula] was kind of a good bridge from us to the patient because… you can [only] build as much rapport with your patient as you can in 12 hours.”* Participant #4
	*“I think the doula…can phrase it even more gently…And since she knew that patient already and the patient trusted her*,* I think the patient was… more willing to answer the doulas questions.”* Participant #4
Doula-Patient Dyad	*“If anybody comes in with a doula*,* they’ve got a great relationship…because they’ve been spending a lot of time with that person… So*,* I respect that.”* Participant #6
	*“Doulas know a lot more [about the patient] on a different level… They can advocate for the patient and as long as the nurse and the doula are working together*,* we can fulfill some of the expectations she may have… the skill set of a doula is meant to support for delivering and I am too*,* so it’s definitely beneficial.”* Participant #1
**Theme 2: Stabilizing Factors that Strengthen Triadic Collaboration**	
Open Communication	*“[The doula is] open to communicate not only with the patient*,* but with the nurse just so the patient’s not getting two different [messages]… The team as a whole understands where she’s [the patient] coming from so we can educate and then maybe even adapt our plan of care.”* Participant #3
Relationship Building and Support	*“If we…had a relationship with the doulas where we were on the same page…I think that would be great for the patients because then they would get that extra support.”* Participant #2
	*“…a doula one time*,* brought in fake candles and some little positive…messages to put on the wall…that was really helpful for that patient.”* Participant #4
	*“They are primarily there to support the emotional well-being of my patient. That’s the biggest…component.”* Participant #4
**Theme 3: Destabilizing Factors that Undermine Triadic Collaboration**	
**Individual-Level Issues**	
Unclear Roles and Scope of Practice	*“There’s no clear-cut definition of what doulas do. They are so hands-on with many aspects of care that it sometimes feels like they’re doing tasks similar to nurses.”* Participant #1
	“*Maybe…the staff communicating with the doula*,* before speaking to the patient like they’re more like a member of the healthcare team and then formulating a plan together… because the doula is always really great at being an advocate for the mom.”* Participant #5
Variable Doula Practices	*“…there are some (doulas) that are very helpful…versus some… that are more hands off…I feel like there’s just different types of doulas.”* Participant #5
**Interpersonal Tensions**	
Advocacy for Unavailable Options	*“Having the doula tell them*,* ‘Pitocin is bad. We should do misoprostol and then your body will actually take over the labor.’ And then the nurse is looking at her toco strip and seeing no contractions with the misoprostol and the doctor being like*,* ‘Hey*,* we need to start Pitocin.‘”* Participant #3
Advocacy Rigidity	*“People go from*,* oh*,* I don’t wanna epidural… And then they’re like…this is really the worst thing… I think I do want that epidural… And…someone always answering. No*,* no*,* they don’t want that… she needed a break from her doula because they were so like*,* no*,* you want your plan*,* you want your birth plan.”* Participant #7
General Mistrust of Doulas	*“…the doula would just negate whatever we said or tell the patient something else and we don’t know what the doula is telling the patient…And we don’t know what education they’re basing their advice off of.”* Participant #2
**System-Level Gaps**	
	Underlying many of the destabilizing experiences described by the nurses was the absence of institutional infrastructure and standardized protocols to support the resolution of individual-level issues or interpersonal tensions.

### Theme 1: The triadic nurse-patient-doula relationship

The primary theme, The Triadic Nurse-Patient-Doula Relationship, describes how nurses consistently perceived doula integration as an interconnected three-party system involving nurse, patient, and doula. Rather than perceiving three separate dyadic relationships (nurse-patient, nurse-doula, doula-patient) operating independently, nurses described a dynamic system where each relationship directly influenced the others. This emerged across all interviews from nurses’ naturalistic descriptions. Participant #5 exemplified this interconnected thinking:

*“…communicating with the doula…before speaking to the patient*,* like they’re more like a member of the healthcare team and then…formulating a plan together and then bringing it up…because the doulas are really great at being advocates for the mom and they’re very knowledgeable on the mom’s plan and sometimes staff can forget that…So if we were all on the same page and then bringing up to the mom together about our plan.”*

**Fig. 1:**
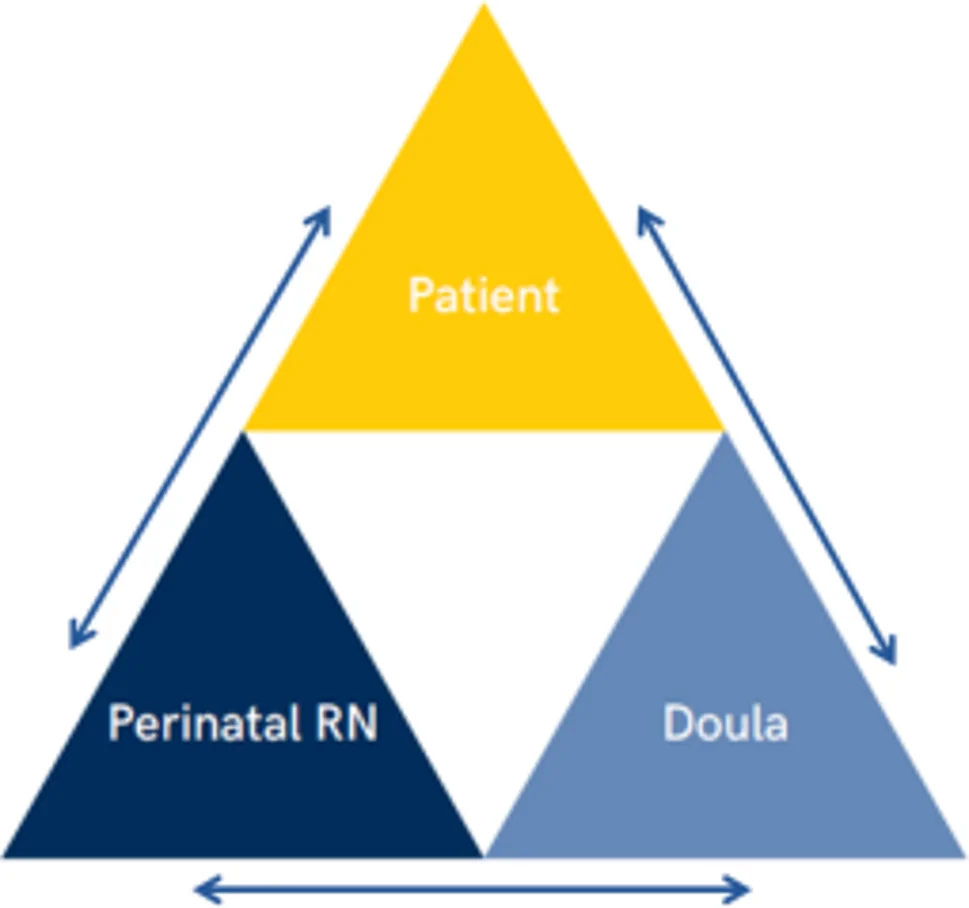
Triadic Nurse-Patient-Doula Relationships in Perinatal Care

This quote clearly shows one of the sequential triadic processes of “nurse > doula > together > patient,” highlighting the interconnection of how the nurse-doula relationship shapes what happens with the patient. (See Fig. 1)

Within this triadic relationship, nurses described distinct dynamics within each dyadic relationship. To present the triadic relationship more clearly, we organize findings by dyad while recognizing their interdependence.

#### Nurse-doula dyad within the triadic relationship

Nurses recognized doulas as addressing care dimensions that time constraints prevented nurses from providing, particularly sustained presence and emotional support. Participant #2 described:

*“*As nurses, we would have to be very well staffed in order to sit at the bedside and rub our patients back for as much as they really needed during labor. We have charting to keep up with; we have maybe more than one patient. We have documenting assessments, talking with the doctors, we’re helping each other. Another nurse is having trouble with their patient; we jump in and help them. So, for a patient to have somebody stay at their bedside and really give them 100% focus*.”*

Recognition of complementary rather than competing roles emerged as a foundation for positive nurse-doula relationships. These positive interactions typically occurred when there was an opportunity for the nurse and doula to establish a relationship, either through previous interactions or care-planning conversations.

#### Nurse-patient dyad within the triadic relationship

Nurses described doulas as facilitating the formation of nurse-patient relationships. Participant #4 termed this a “bridge”: *“She [the doula] was kind of a good bridge from us to the patient because… you can [only] build as much rapport with your patient as you can in 12 hours.”* Doulas’ pre-existing relationships with patients created a relational foundation that nurses could leverage to support rapport development within their shift timeline.

Nurses recognized doulas could *“phrase things more gently”* or facilitate difficult conversations. Participant #4 explained:

*“*I think the doula…can phrase it even more gently…And since she knew that patient already and the patient trusted her, I think the patient was… more willing to answer the doulas questions*.”*

This quote demonstrates how doulas’ pre-existing relationships and communication styles can enhance nurses’ ability to gather information and provide patient-centered care. 

#### Doula-patient dyad within the triadic relationship

Nurses identified the doula-patient relationship through specific behavioral indicators: physical closeness and touch, patients’ preferential attention to doula input during decision-making, and shared communication patterns suggesting extended interaction history. Participant #6 explained: *“If anybody comes in with a doula*,* they’ve got a great relationship…because they’ve been spending a lot of time with that person… So*,* I respect that.”* This recognition of the doula-patient relationship was grounded in observable markers of established rapport. Nurses respected these existing bonds as legitimate, patient-chosen support relationships deserving of professional accommodation.

The doula-patient relationship served multiple functions that nurses recognized as valuable: emotional support complementing medical care, advocacy reflecting patients’ birth preferences, and continuity bridging prenatal through intrapartum care. Participant #1 stated:

*“*Doulas know a lot more [about the patient] on a different level… They can advocate for the patient and as long as the nurse and the doula are working together, we can fulfill some of the expectations she may have… the skill set of a doula is meant to support for delivering and I am too, so it’s definitely beneficial*.”*

By clearly distinguishing doula roles from medical tasks, this quote highlights how nurses understood doulas as providing unique, complementary value in patient care. However, when doulas’ advocacy conflicted with evolving clinical needs, nurses sometimes perceived this as compromising necessary care adaptations.

#### Summary of triadic nurse-patient-doula relationship

Across all interviews, nurses perceived doula integration as an interconnected system where each relationship influenced the others. The quality of the nurse-doula relationship affected the nurse-patient relationship, and vice versa. Optimal patient care coordination required the nurses to have a working understanding and consideration of the doula-patient relationship dynamics. The triadic relationship’s quality depended on specific stabilizing and destabilizing factors.

### Theme 2: Stabilizing factors that strengthen triadic collaboration

The second theme, Stabilizing Factors that Strengthen Triadic Collaboration, identified factors that strengthened triadic collaboration and enhanced the feasibility of doula integration. Two primary mechanisms emerged: (1) Open communication – practices establishing shared understanding, clarifying roles, and preventing conflicts through dialogue; and (2) Relationship building and support – factors creating positive regard, mutual respect, and complementary role perceptions among all three parties. See Table 3 for participants’ characteristics.

#### Open communication

Nurses consistently identified proactive communication as essential for triadic collaboration. Three practices emerged: introductions clarifying each person’s role, explicit boundary setting preventing coordination conflicts, and collaborative care planning aligning all parties’ goals. Participant #3 described an ideal communication structure:

*“[The doula is] open to communicate not only with the patient*,* but with the nurse just so the patient’s not getting two different [messages]… The team as a whole understands where she’s [the patient] coming from so we can educate and then maybe even adapt our plan of care.”*

This quote captures the essence of open communication by demonstrating how structured dialogue between all three parties creates unified care goals. Effective communication required bilateral coordination across all three dyads: nurse-doula (to establish coordination), nurse-patient (to maintain primary care coordination), and doula-patient (to respect established bonds).

#### Relationship building and support

This category encompassed factors building positive regard, trust, and role appreciation across the triad. Three patterns emerged: pre-existing relationships that provided relational foundation, valued doula contributions nurses couldn’t replicate, and role harmonizing positioning doula and nurse contributions as synergistic rather than competitive.

Nurses respected the established patient-doula bonds. Participant #2 stated:

*“*If we…had a relationship with the doulas where we were on the same page…I think that would be great for the patients because then they would get that extra support*.”*

This respect contrasted with potential territorial professional responses, indicating nurses’ patient-centered orientation valued continuity even when it meant sharing influence.

Nurses appreciated doula practices enhancing patient experience in ways nurses lacked time or skills to provide. Participant #4 described:

*“…a doula one time*,* brought in fake candles and some little positive…messages to put on the wall…that was really helpful for that patient.”*

Nurses who recognized distinct doula contributions expressed positive attitudes. Participant #4 continued by saying: *“They are primarily there to support the emotional well-being of my patient. That’s the biggest…component.”* This quote specifically names emotional support as the primary doula contribution, clearly distinguishing doula roles from medical tasks and highlighting their unique value in patient care.

### Theme 3: Destabilizing factors that undermine triadic collaboration

The third theme, Destabilizing Factors that Undermine Triadic Collaboration, identified factors undermining triadic collaboration and creating implementation barriers. Analysis revealed destabilizing factors at multiple levels: (1) Individual-level issues including unclear roles, scope confusion, and variable doula practices; (2) Interpersonal tensions including advocacy conflicts and mistrust; and (3) System-level gaps including lack of institutional infrastructure and standardization. See Table 3 for participant characteristics.

#### Individual-level issues

##### Unclear roles and scope of practice

Nurses expressed fundamental uncertainty about doulas’ scope of practice: what doulas were trained to do, what they were permitted to do, and what they actually did in practice. Participant #1 stated:

*“There’s no clear-cut definition of what doulas do. They are so hands-on with many aspects of care that it sometimes feels like they’re doing tasks similar to nurses.”*

This ambiguity created confusion about delegation, supervision, and accountability.

The result of the nurse-doula dyad revealed that role ambiguity and hierarchical uncertainty characterized nurses’ experiences with doulas. Unlike other team members whose scopes of practice and communication protocols are institutionally defined, nurses noted the doula role remained ambiguous. This ambiguity created care coordination challenges. Participant #5 articulated this structural challenge:

*“Maybe…the staff communicating with the doula*,* before speaking to the patient like they’re more like a member of the healthcare team and then formulating a plan together… because the doula is always really great at being an advocate for the mom.”*

This description reveals the nurses’ desire to include doulas as “*member(s) of the healthcare team*” but uncertainty about procedural mechanisms for engagement. 

##### Variable doula practices

Compounding scope ambiguity was variability in how individual doulas enacted their roles. Participant #5 noted:

*“…there are some (doulas) that are very helpful…versus some… that are more hands off…I feel like there’s just different types of doulas.”*

This inconsistency prevented nurses from developing stable expectations based on prior experiences; each encounter required fresh role negotiation.

#### Interpersonal tensions

##### Advocacy for unavailable options

Destabilizing factors emerged when doulas advocated for care options that nurses and physicians deemed medically unavailable or inappropriate. This created tensions: patient expectations were raised for unavailable options, nurses’ clinical judgment was questioned, and shared care planning was eroded. Participant #3 provided an example:

*“Having the doula tell them*,* ‘Pitocin is bad. We should do misoprostol and then your body will actually take over the labor.’ And then the nurse is looking at her toco strip and seeing no contractions with the misoprostol and the doctor being like*,* ‘Hey*,* we need to start Pitocin.‘”*.

##### Advocacy rigidity

Nurses described situations where the doulas’ advocacy rigidity manifested in two related ways: when doulas maintained advocacy rigidity against clinically indicated changes, and when doulas maintained rigid advocacy against the patient’s own evolving preferences. Participant #7 described:

*“*People go from, oh, I don’t wanna epidural… And then they’re like…this is really the worst thing… I think I do want that epidural… And…someone always answering. No, no, they don’t want that… she needed a break from her doula because they were so like, no, you want your plan, you want your birth plan*.”*

This illustrated how patients’ preferences changed during labor, yet the doula continued advocating for the original birth plan despite changing needs.

##### General mistrust of doulas

Nurses expressed preexisting mistrust of doulas, which shaped initial interactions and created barriers to collaborative relationship formation. Concerns included doulas’ conduct during unsupervised patient interactions and uncertainty about the knowledge base informing doula advice. Participant #2 articulated nurses specific mistrust concerns:

*“*…the doula would just negate whatever we said or tell the patient something else and we don’t know what the doula is telling the patient…And we don’t know what education they’re basing their advice off of*.”*

This quote reveals multiple concerns; concerns about doulas undermining a nurses’ authority, uncertainty about doula-patient communication content when nurses aren’t present, and a lack of transparency about doulas’ educational preparation. This mistrust appears to stem from structural factors rather than solely interpersonal mistrust regarding doula conduct during patient interactions. 

#### System-level gaps

##### Lack of Institutional Infrastructure and Standardization

Underlying many destabilizing experiences was the absence of formalized institutional integration defining doula roles, scope, training expectations, and coordination protocols. Without this infrastructure and standardization, nurses experienced persistent role ambiguity that created multiple challenges including delegation confusion, supervision uncertainty, and accountability concerns. This structural gap meant that individual-level issues and interpersonal tensions had no institutional mechanisms for clarification or resolution.

## Discussion

We conducted the pre-implementation needs assessment to examine perinatal nurses’ perspectives on doula integration. The findings revealed that primarily white nurses with limited exposure to doulas, like those in our study, fundamentally perceive doula integration through triadic relationships, a relational perspective that emerged consistently across all interviews. As critical stakeholders whose perspectives directly determine daily implementation success [13], nurses make real-time decisions when integrating doula support into clinical workflows, navigating situations where doula advocacy conflicts with medical recommendations, and coordinating care planning conversations. Without nurses’ buy-in and active collaboration, doula programs cannot function effectively regardless of organizational support [9, 18]. Rather than being resistant to doula integration, our results indicate that nurses seek clarity about doula roles and transparent communications about doula training and expectations. They also seek assurance that doulas will work collaboratively as nursing allies who provide complementary emotional support rather than competing with or substituting for nursing care.

Previous research examining interprofessional relationships in maternity care has primarily focused on dyadic partnerships treating these as isolated interactions [19, 20]. For example, studies of nurse-doula relationships have identified communication barriers and role confusion but analyzed these as two-party dynamics without considering how patient relationships influence professional interactions [20]. Similarly, research on patient-provider relationships often examines the nurse-patient or physician-patient dyad in isolation [21]. Our conceptualization of triadic relationships challenges the traditional dual dynamic by demonstrating that these relationships are fundamentally interconnected in the context of doula integration. Nurses in our study spontaneously described their work through triadic language (“*We all have the same goal*,” “*The team as a whole*,” “*Working together*”) indicating that they naturally perceive doula integration as a three-party system. Our findings suggest that merely optimizing any single dyad (e.g., improving nurse-doula communication) may be insufficient as it could destabilize another relationship in the triad (e.g., if it creates patient confusion about conflicting advice).

Our findings indicate that stabilizing factors (communication, relationship-building) and destabilizing factors (ambiguity, mistrust, lack of institutional infrastructure) operate at the intersection of individual, interpersonal, and system levels, suggesting that successful implementation requires multi-level intervention strategies addressing all three simultaneously [7]. Our results emphasize that when interconnected relationships function well with open communication, role clarity, and mutual respect, patients benefit from coordinated support addressing both medical safety and emotional well-being, which Marshall and Kozhimannil [22] describe as requiring “continual dialogue” and deliberate respect for the doula’s nonclinical scope of practice. This is particularly important for vulnerable populations experiencing health disparities who need the doula’s continuous presence and pre-existing patient relationship to bridge gaps that time-constrained nursing care may not be able to address [22]. However, our results found that when relationships are unstable due to role ambiguity or interpersonal tensions, patients who would benefit most from doula support may experience fragmented care and increased stress during labor thus making optimization of triadic relationships not merely an implementation element but a health equity imperative ensuring all patients receive coordinated, respectful, culturally responsive maternity care.

Our stabilizing and destabilizing findings complement but extend beyond previous implementation research that has emphasized organizational and structural barriers to doula integration [3, 23, 24]. Specifically, recent scoping reviews by Kang et al. [23] and Alvarado et al. [24] identified key system-level barriers including lack of Medicaid reimbursement, inconsistent credentialing standards, workforce capacity limitations, and absence of hospital policies supporting doula programs. Similarly, Wyand et al.‘s [3] environmental scan documented significant regional variation and inconsistent program structures. Consistent with these findings, Marshall and Kozhimannil [22] observe that even as Medicaid doula coverage expands, adding doula care as a covered benefit does not necessarily ensure access due to persistent implementation barriers across multiple levels.

While these organizational factors are undeniably important, our findings reveal that addressing them alone may be insufficient for successful implementation. Nurses in our study worked at a hospital actively planning doula program implementation, thus organizational readiness was established. Yet they still identified substantial interpersonal challenges that could undermine daily program functioning. This suggests that doula integration requires multi-level interventions addressing (1) Individual-level issues including unclear roles, scope confusion, and variable doula practices; (2) Interpersonal tensions including advocacy conflicts and mistrust; and (3) System-level gaps including lack of institutional infrastructure and standardization. Implementation strategies that focus exclusively on organizational readiness without attending to triadic relationship dynamics may successfully launch doula programs but encounter barriers to carrying them through and sustaining them when bedside tensions emerge.

Furthermore, we cautiously interpret our results because the nurses in our study were primarily white and had limited prior experience working with doulas. In future research it is important to seek the perspective of ethnically diverse nurses more explicitly, their voices may enrich multilevel implementation strategies. Additionally, those nurses who have more experience working directly with doulas may have further knowledge to share that could refine or alter the triadic framework.

### Implications for nursing practice

#### For bedside nurses

For bedside nurses, our findings provide guidance to facilitate productive collaboration with doulas. Understanding the three relational factors (trust transfer, role coordination, and advocacy alignment) can help nurses proactively strengthen collaboration at the bedside.

Recommendations include initiating structured introductions that explicitly discuss each person’s role, establish communication preferences, and identify the patient’s goals for birth support. This practice addresses role coordination by reducing ambiguity in scope of practice, decision-making authority, and communication protocols before uncertainty affects patient care planning. Additionally, nurses can leverage trust transfer by acknowledging the pre-existing patient-doula relationship creates a foundation of trust that can facilitate, rather than hinder, nurse-patient rapport development. We recommend nurses leverage the doulas’ continuous presence and established patient relationships strategically by asking the doula to help support the patient during times when they need to be out of the room. This practice could enhance care coordination, rather than creating competition.

Lastly, we encourage nurses to address advocacy alignment proactively. This means that when conflicts arise, addressing them outside the patient’s room may help to maintain a united team presence. In this way, nurses and doulas frame disagreements as opportunities for collaborative problem-solving. 

#### For nurse leaders and managers

Nurse leaders planning doula program implementation should recognize that organizational readiness alone is insufficient [9–12]. Our findings revealed three specific relational mechanisms that required targeted intervention: trust transfer, role coordination, and advocacy alignment. Implementation strategies should address each mechanism explicitly.

To facilitate trust transfer, we recommend developing standardized doula orientation programs that include job shadowing with nurses, explicit discussion of scope of practice boundaries, and collaborative protocol development. By creating opportunities for nurses and doulas to work together and build relationships, you can help nurses understand how patients’ pre-existing doula relationships can enhance (not undermine) nurse-patient rapport if properly leveraged. Some suggestions include: interprofessional simulation exercises [25], joint training sessions on physiologic birth support [13], or informal meet-and-greet events [26].

To strengthen role coordination, we recommend establishing clear institutional policies that define the doula role, team communication expectations, and escalation procedures when conflicts arise [27]. Proactively addressing ambiguity in scope of practice, decision-making authority, and communication protocols may help reduce uncertainty that affects patient care planning and team functioning. Proactive communication protocols also help to encourage advocacy alignment. Adding mechanisms for ways to navigate situations when doula advocacy for birth preferences may conflict with evolving clinical needs can assist nurses and doulas in aligning advocacy efforts with clinical realities before conflicts arise.

Most importantly, our results show that involving bedside nurses in all stages of program planning, from developing policies to selecting implementation strategies to evaluating outcomes is critical. Nurses who feel ownership over doula integration are more likely to champion it with colleagues. Consider designating nurse “doula champions” who can mentor peers, troubleshoot challenges, and provide feedback to program leadership [28]. 

#### For nursing educators

Nursing education must address explicitly doula integration and interprofessional collaboration in maternity care curricula. Research documents that territorial conflicts between labor nurses and doulas are not uncommon in clinical practice; nurses do not always understand the doula’s role, and some nurses have been described as adversarial in their dealings with doulas [20]. These behaviors have direct implications for nursing education: Curricula that explicitly differentiate and affirm the complementary roles of nurses and doulas, where nurses coordinate evidence-based clinical care while the doula provides continuous emotional and physical support [12, 29]. This approach may help disrupt these dynamics before students enter clinical practice and align with the Association of Women’s Health, Obstetric and Neonatal Nurses (AWHONN) [30] position that nurses should engage in respectful collaborative relationships with doulas as key members of the interprofessional birthing team, and with growing evidence that nurse-doula collaboration contributes to improved maternal outcomes [22].

Academic nurse educators should teach communication strategies for addressing disagreements professionally and in a patient-centered manner. Exposure to doula collaboration during nursing school can normalize it as part of professional practice rather than as a threatening addition encountered only after graduation [29, 31].

### Study strengths and limitations

This study has several notable strengths. First, our study contributes to the literature by focusing specifically on nurses’ perspectives and addressing a critical gap in doula implementation research. While previous research has examined patient outcomes and doula perspectives [20], less attention has been paid to nurses as key stakeholders who serve as gatekeepers determining daily program functioning at the bedside. Second, the pre-implementation timing enabled us to capture nurses’ perspectives during the critical planning phase when attitudes significantly influence adoption success [7, 32] before implementation challenges obscure initial concerns and expectations. This proactive approach aligns with implementation science best practices emphasizing stakeholder engagement before program launch [7, 20] which is particularly critical for doula integration where establishing trust and role clarity between interprofessional team members directly impacts collaboration and patient outcomes.

Study rigor was enhanced through two complementary approaches: (1) rigorous qualitative methods including trustworthiness procedures, team-based consensus coding, and external auditing, and (2) an interdisciplinary research team that combined insider knowledge of perinatal nursing practice with analytical distance necessary for qualitative inquiry, together ensuring findings accurately represent nurses’ perspectives while strengthening both data collection and interpretation.

Finally, several limitations should be considered when interpreting these findings. First, this single-site study at an urban academic medical center preparing to launch a doula program may not fully represent nurses’ perspectives in settings with established programs, different organizational cultures, or community (versus academic) hospital contexts. Findings may be most transferable to similar academic medical centers with planned doula implementation and Magnet designation. Second, participants had limited doula exposure (71.4% rarely worked with doulas, 28.6% sometimes worked with them), meaning perspectives reflect anticipated concerns and hypothetical scenarios rather than actual experience. While appropriate for pre-implementation assessment, this means the themes identified in this study may reflect perceived barriers and facilitators versus ones identified by nurses with more consistent experience working with doulas. Third, our study captured the perspectives of primarily white nurses. A complete understanding of triadic dynamics requires interviews with ethnically diverse nurses, as well as doulas and patients to identify convergence and divergence in how each party experiences these relationships. Fourth, volunteer bias may mean participants have stronger interest or more definitive opinions (positive or negative) about doulas than non-participants. Finally, the small sample size (*n* = 7) limits generalizability, though data saturation was achieved within this homogeneous population, indicating thematic sufficiency for this pre-implementation context.

### Future research directions

Future research should pursue several directions to build on these findings. First, longitudinal, multi-site descriptive studies are needed to confirm whether the triadic relationship holds across diverse settings (community hospitals, birth centers, academic medical centers), organizational cultures, patient populations, and geographic regions. Multi-stakeholder perspective studies including parallel interviews with a diverse panel of nurses, doulas, patients, and physicians would provide comprehensive understanding of how each party experiences triadic dynamics, identifying convergence and divergence in perspectives. Researchers should also investigate how contextual factors impact triadic relationship dynamics. Evidence already indicates that doula program maturity, defined as the percentage of births with doula attendance, is a significant predictor of nurses’ attitudes toward doulas, as are workload and staffing conditions [12, 33]. The complexity of labor and delivery organizational cultures further shapes whether interprofessional interventions are realistically implemented or sustained over time [34] underscoring the need to explicitly measure how program maturity, staffing ratios, and patient acuity impact triadic dynamics across clinical contexts.

Second, doula integration intervention studies should focus on implementing multi-level strategies and using rigorous evaluation to assess feasibility, acceptability, safety, and impact. In a study with a mixed-methods trial of TeamBirth, a communication and shared decision-making intervention across four community hospitals, offers a methodological exemplar for this approach [34]. They used detailed study protocols, cross-referenced all measures against the Consolidated Framework for Implementation Research (CFIR), to explicitly assess feasibility, acceptability, and safety before proceeding to effectiveness testing [34]. Post-implementation evaluation research should examine how nurses’ perspectives evolve with sustained doula program experience.

Our pre-implementation data identified three critical elements requiring advance attention: education about doula roles and training, relationship development to establish trust, and clear role-delineation. To assess implementation progress, these elements should be tracked longitudinally to determine whether (1) anticipated concerns (e.g. role ambiguity, trust-building challenges, communication barriers) materialize or resolve with experience, (2) advanced preparation strategies (e.g. institutional policies, communication guidelines) mitigate these concerns, and (3) new unanticipated challenges emerge that were not apparent in the pre-implementation assessment. It is essential to understand how doula integration programs, including triadic relationship-focused implementation, will impact perinatal outcomes.

## Conclusion

The contribution of our pre-implementation needs assessment revealed that nurses, the primary care coordinators at the bedside, consistently perceive doula integration through a triadic relationship involving interconnected nurse-patient-doula dynamics. Successful integration depends on specific stabilizing factors (open communication, role clarity, relationship building) while destabilizing factors (scope ambiguity, advocacy conflicts, mistrust, lack of institutional infrastructure and standardization) undermine collaboration and threaten program sustainability. These findings provide guidance for intervention design: structured communication protocols, standardized doula orientation emphasizing role complementarity, collaborative birth planning mechanisms, and institutional policies clarifying scope of practice. Future study should include racially and ethnically diverse nursing perspectives related to implementation strategies.

## Data Availability

Data sharing is not applicable due to the approved IRB protocol. Please contact corresponding author (Leanne Burke @ Ltburke@uci.edu) for inquiry about data analyzed in this study.

## Abbreviations

AWHONN: Association of Women’s Health Obstetric and Neonatal Nurses
CFIR: Consolidated Framework for Implementation Research
